# Research on Roll Attitude Estimation Algorithm for Precision Firefighting Extinguishing Projectiles Based on Single MEMS Gyroscope

**DOI:** 10.3390/s25216721

**Published:** 2025-11-03

**Authors:** Jinsong Zeng, Zeyuan Liu, Chengyang Liu

**Affiliations:** School of Mechanical and Power Engineering, Zhengzhou University, Zhengzhou 450001, China; zengjs@zzu.edu.cn (J.Z.); lcy2001@gs.zzu.edu.cn (C.L.)

**Keywords:** precision firefighting projectiles, roll attitude calculation, Fourier transform, IIR filter, single-sensor attitude measurement

## Abstract

The accurate acquisition and real-time calculation of the attitude angle of precision firefighting extinguishing projectiles are essential for ensuring stable flight and precise extinguishing agent release. However, measuring the roll attitude angle in such projectiles is challenging due to their highly dynamic nature and environmental disturbances such as fire smoke, high temperature, and electromagnetic interference. Traditional methods for measuring attitude angles rely on multi-sensor fusion schemes, which suffer from complex structure and high cost. This paper proposes a single-gyro attitude calculation method based on micro-electromechanical inertial measurement units (MIMUs). This method integrates Fourier transform time-frequency analysis with a second-order Infinite Impulse Response (IIR) bandpass filtering algorithm optimized by dynamic coefficients. Unlike conventional fixed-coefficient filters, the proposed algorithm adaptively updates filter parameters according to instantaneous roll angular velocity, thereby maintaining tracking capability under time-varying conditions. This theoretical contribution provides a general framework for adaptive frequency-tracking filtering, beyond the specific engineering case of firefighting projectiles. Through joint time-frequency domain processing, it achieves high-precision dynamic decoupling of the roll angle, eliminating the dependency on external sensors (e.g., radar/GPS) inherent in conventional systems. This approach drastically reduces system complexity and provides key technical support for low-cost and high-reliability firefighting projectile attitude control. The research contributes to enhancing the effectiveness of urban firefighting, forest fire suppression, and public safety emergency response.

## 1. Introduction

With the breakthrough in Micro Inertial Measurement Unit (MIMU) technology, the autonomous navigation capability of firefighting extinguishing projectiles has become a core support for the precision fire extinguishing system in modern fire scenes [1]. Due to the azimuth-free loading mode of firefighting extinguishing projectiles and various disturbances they suffer from inside and outside the barrel, the roll angle cannot be initially set [2]. The real-time and accurate calculation of roll attitude parameters not only determines the flight stability of extinguishing projectiles, but also serves as a prerequisite for realizing trajectory correction and terminal precise extinguishing control [3]. Although traditional multi-sensor fusion attitude measurement methods (such as GPS/INS integration, radar assistance, etc.) can calculate the attitude, their high-precision requirements for multi-source information synchronization lead to increased system redundancy, exposure of electromagnetic characteristics, and reduced fire scene adaptability. Especially in high-dynamic and strong confrontation environments, the unreliability of external navigation signals directly threatens the reliability of the control system in fire environments [4]. Therefore, constructing an autonomous air alignment algorithm based on a single MIMU is not only a practical need to reduce system complexity and cost, but also a key path to improve the robustness and concealment of firefighting device systems.

Integrated attitude measurement methods usually improve accuracy and robustness by fusing complementary sensor information [5]. For example, the short-term dynamic response advantage of inertial devices is used to make up for the long-term stability deficiency of satellite navigation [6], or the geomagnetic characteristic of no accumulated error is used to correct the drift of inertial devices [7,8,9]. However, such methods rely on the collaboration of multiple sensors, and have problems such as complex systems, high costs, and weak anti-interference ability. Experiments by Yang Shu [10] show that GNSS signals are susceptible to multipath effects (reflection and scattering) in urban environments, resulting in elevation measurement errors; at the same time, poor satellite geometric distribution (such as overly concentrated constellations) will further increase the positioning error in the elevation direction. The experimental results show that the GNSS motion trajectory fluctuates significantly in the elevation direction, with the difference between the highest and lowest points reaching about 8 m. Although the 5G enhancement scheme proposed by Zhang Baoting [11] effectively improves the timeliness and stability of system data transmission, it requires additional integration of 5G communication modules, leading to increased system complexity. Cao Xiang [12] conducted an analysis on the global positioning performance of the BDS-3/GPS integrated navigation system, and the results showed that in complex observation environments, the average number of visible satellites of the integrated system decreases significantly, and the mean value of GDOP (Geometric Dilution of Precision) increases obviously, which is difficult to meet the environmental requirements for projectile launch. Gao Lizhen [13] constructed a state equation based on the projectile motion model, took geomagnetic/gyro information as the measurement quantity, and optimized the real-time performance of the filtering process and the adaptive estimation performance through sequential filtering and adaptive algorithms. The single-gyro attitude measurement method proposed by Yang Qifan’s team [14] provides a new idea for attitude measurement technology, but the Finite Impulse Response (FIR) filter in this method has a large amount of calculation, and the accuracy of its filter frequency solution still needs to be improved.

In international research, many scholars have also explored IMU/GNSS integration and error modeling. Fukuda and Kubo proposed a bias estimation method for low-cost IMUs, which improves the attitude stability of INS/GPS/gyrocompass systems [15]. Pang et al. designed an IMU/GPS integrated navigation method based on the Adaptive Unscented Kalman Filter (AUKF), and applied it to the three-dimensional positioning of forest rescue personnel, verifying its robustness [16]. Nader et al. proposed a loosely coupled GPS/IMU integration scheme based on two cascaded Kalman filter stages, which significantly enhanced the stability of attitude estimation [17]. Ismail and Abdelkawy presented a hybrid error modeling approach for MEMS-IMUs, effectively compensating for systematic errors of low-cost inertial devices in GPS/INS integration [18]. In addition, Gao et al. systematically evaluated the impact of IMU grades on the accuracy of BDS+GPS PPP/INS tightly coupled integration, and highlighted the limitations of low-cost IMUs in high-precision navigation applications [19].

Meanwhile, some researchers have started to explore the potential of single-sensor or low-cost MEMS-based attitude estimation. Deng Zilong [20] proposed an FLL-PLL combined roll angle detection method based on roll modulation information in GPS signals. Wu et al. [21] proposed a single-antenna GNSS/MEMS fusion structure, which integrates TDCP/TDPR techniques with multi-strategy quality control, achieving a roll angle estimation accuracy of approximately 1.28°. Zhou et al. [22] introduced a hierarchical decoupled attitude estimation algorithm (HDAEA), which maintained high-accuracy UAV attitude estimation even under strong magnetic interference and highly dynamic conditions. Dang and Suh [23] investigated a single inertial-sensor-based attitude estimation method and improved roll angle accuracy through velocity-aided observations.

To address the issues of heavy reliance on multi-sensor fusion, increased system complexity and cost, and the difficulty of ensuring the real-time performance and accuracy of attitude estimation under weak-signal or strong-interference environments, this paper proposes an innovative time–frequency analysis and filtering fusion algorithm. By constructing a dynamic error model of the rotating extinguishing projectile, the method couples time–frequency domain feature extraction with adaptive filter optimization. This approach not only guarantees the accuracy of attitude estimation, but also significantly reduces computational complexity and enhances stability in strong interference environments. The proposed method provides a new pathway for achieving high-precision autonomous attitude determination using a single MIMU.

In summary, the contributions of this paper are threefold:

1. A roll attitude estimation framework is established based on a single MEMS gyroscope, incorporating installation error modeling that enables practical deployment on low-cost projectiles.
2. A time–frequency adaptive IIR filtering algorithm is developed to track the time-varying roll angular rate and separate roll-related components from the measured signal.
3. The proposed method is validated through both simulation and turntable experimentation, demonstrating its accuracy and robustness under dynamic motion conditions.

The remainder of this article is organized as follows:

Section 2 presents the methodology and theoretical analysis, including the installation error modeling.

Section 3 introduces the adaptive filtering design.

Section 4 describes the simulation and experimental validation.

Section 5 concludes the paper with remarks and future improvements.

## 2. Methodology and Theoretical Analysis

### 2.1. Overall Design Concept of the Method

**Figure 1:**
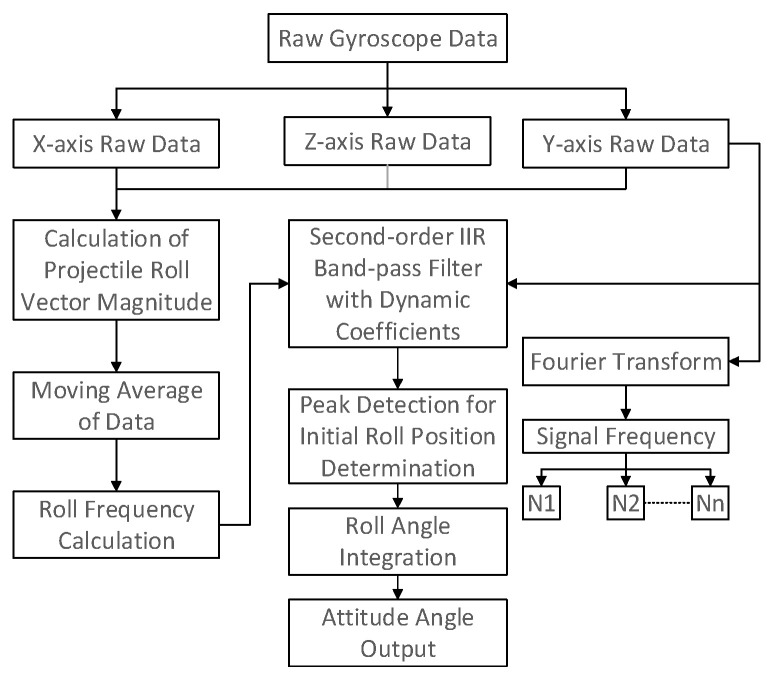
Overall Block Diagram of the Proposed Method (this diagram illustrates the overall framework of the roll attitude estimation algorithm, showing the signal flow from sensor measurement to final correction).

Figure 1 shows the overall design block diagram of the method proposed in this paper, where N1~Nn represent the amplitudes of the signal obtained after Fourier transform (arranged from low frequency to high frequency in sequence).

The method proposed in this paper mainly includes the following 3 steps:

1. Data analysis and preprocessing: Calculate the modulus of the original gyroscope data to obtain the estimated value of the extinguishing projectile’s roll angular velocity, perform moving average calculation on the estimated value of the extinguishing projectile’s roll angular velocity, and solve the roll frequency to prepare for subsequent filtering processing.
2. Data filtering: Perform filtering processing in the frequency domain. Considering that the change in roll angular velocity will cause frequency changes, this study combines angular velocity to perform dynamic coefficient second-order IIR band-pass filtering.
3. Attitude angle calculation: Perform peak detection on the filtered data to determine the roll position of the extinguishing projectile. Calculate the roll attitude angle by combining the integral of the actual roll angular velocity of the extinguishing projectile, and output the roll attitude angle for the control of the extinguishing projectile.

### 2.2. Principle Analysis of Single-Gyro Attitude Measurement

In practice, due to the existence of installation errors, the three axes of the gyroscope cannot be completely aligned with the three axes of the extinguishing projectile body [24]. This causes the roll angular velocity to project onto the plane perpendicular to the extinguishing projectile’s roll axis, and the magnitude of the actual roll angular velocity of the extinguishing projectile is much larger than that of the pitch and yaw angular velocities, which reduces the reliability of the data output by the gyroscope. As a result, single-sensor attitude measurement is rarely used in practice.

**Figure 2:**
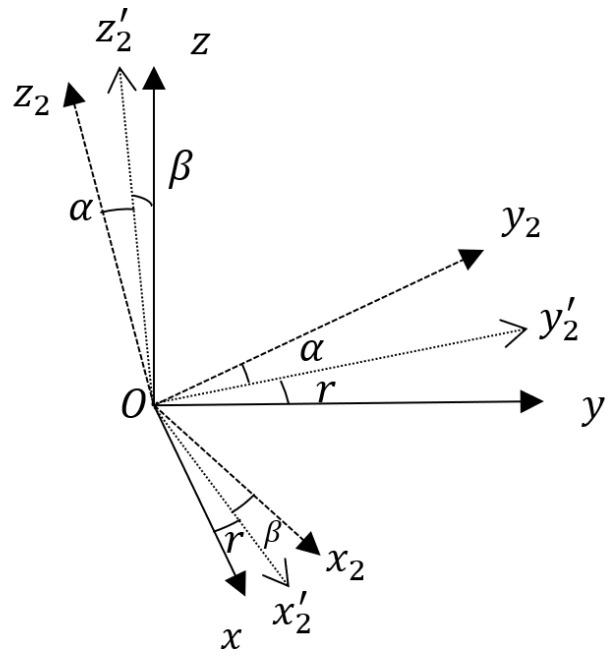
Rotation between the reference and Body Coordinate Systems (This schematic defines the relationship between the reference and body coordinate systems used for attitude computation).

**Figure 3:**
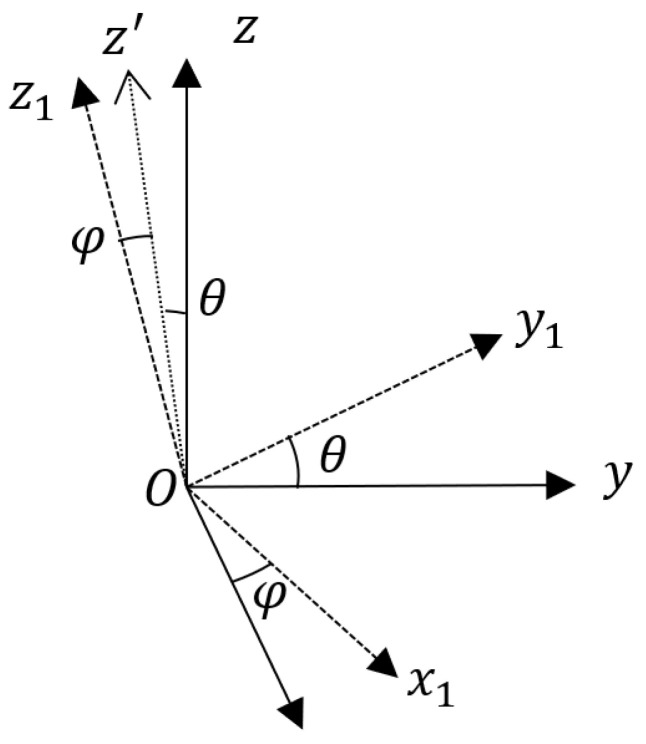
Rotation between the Body and Gyroscope Coordinate Systems (This figure shows the installation error model between the body frame and gyroscope frame, which is considered in the theoretical derivation).

Define the reference coordinate system $Ox_{2}y_{2}z_{2}$, with the origin O is located at the extinguishing projectile’s center of mass. The $Oz_{2}$ axis lies in the local horizontal plane and points to the target axis; the $Ox_{2}$ axis is perpendicular to the local horizontal plane, with the downward direction as the positive direction; the $Oy_{2}$ axis is perpendicular to the $Ox_{2}z_{2}$ plane, forming a right-handed rectangular coordinate system with the $Ox_{2}$ and $Oz_{2}$ axes. This coordinate system translates with the extinguishing projectile’s center of mass, while its three axes remain fixed in orientation. Define the extinguishing projectile body coordinate system Oxyz, with the origin O at the extinguishing projectile’s center of mass. The Oz axis coincides with the extinguishing projectile’s longitudinal axis, with the direction pointing to the warhead as the positive direction; the Ox axis lies in the extinguishing projectile’s longitudinal symmetry plane, with the zero position downward as the positive axis; the Oy axis is perpendicular to the plane, forming a right-handed coordinate system with the Ox and Oz axes. Define the yaw angle as α, the pitch angle as β, and the roll angle as r. According to Euler’s theorem, the reference coordinate system $Ox_{2}y_{2}z_{2}$ can be transformed into the projectile body coordinate system Oxyz through a sequence of rotations: first, rotate the reference coordinate system around the $Ox_{2}$ axis by α to obtain coordinate system $Ox_{2}y_{2}^{\prime }z_{2}^{\prime }$; then rotate $Ox_{2}y_{2}^{\prime }z_{2}^{\prime }$ around the $Oy_{2}^{\prime }$ axis by β to obtain coordinate system $Ox_{2}^{\prime }y_{2}^{\prime }z$; and finally rotate $Ox_{2}^{\prime }y_{2}^{\prime }z$ around the Oz axis by r, resulting in the extinguishing projectile body coordinate system Oxyz (as shown in Figure 2). Define the gyroscope coordinate system $Ox_{1}y_{1}z_{1}$, with the origin $Ox_{1}$ at the extinguishing projectile’s center of mass. Due to installation errors, the three axes of the gyroscope coordinate system do not align with those of the extinguishing projectile body coordinate system. The gyroscope coordinate system $Ox_{1}y_{1}z_{1}$ can be obtained from the projectile body coordinate system Oxyz through the following rotations: first, rotate the projectile body coordinate system Oxyz around the Ox axis by θ to obtain coordinate system $Oxy_{1}z^{\prime }$; then rotate $Oxy_{1}z^{\prime }$ around the $Oy_{1}$ axis by φ, resulting in the gyroscope coordinate system $Ox_{1}y_{1}z_{1}$ (as shown in Figure 3). Here, θ and φ are the installation error angles between the Oy and $Oy_{1}$ axes, and between the Ox and $Ox_{1}$ axes, respectively. The installation deviation of the Oz axis is mathematically indistinguishable from the roll variable and is thus categorized as an unobservable term, which cannot be directly modeled or corrected through real-time solution processes. However, it should be noted that this error is a fixed systematic deviation—its pre-elimination via offline calibration suffices to meet engineering error requirements. After two rotations, none of the three axes of the two coordinate systems remain aligned, a scenario consistent with actual installation conditions. Thus, excluding this deviation from the modeling is theoretically justified.

At a certain moment, the three-axis angular velocity of the extinguishing projectile in the reference coordinate system $Ox_{2}y_{2}z_{2}$ is:

(1) $$\omega_{2}={\left[\omega_{x_{2}},\omega_{y_{2}},\omega_{z_{2}}\right]}^{T}$$

At this time, the three-axis angular velocity of the extinguishing projectile in the extinguishing projectile body coordinate system Oxyz can be obtained through rotation matrix transformation:

(2) $$\omega =R_{2}\cdot \omega_{2}$$

In Equation (2), $R_{2}$ is the rotation matrix from the reference coordinate system to the extinguishing projectile body coordinate system, which is composed of the product of three rotation matrices:

(3) $$R_{2}=R_{z}\left(r\right)\cdot R_{y}\left(\beta \right)\cdot R_{x}\left(\alpha \right)=\left[\begin{array}{ccc} cos\;r & -sin\;r & 0\\ sin\;r & cos\;r & 0\\ 0 & 0 & 1 \end{array}\right]\cdot \left[\begin{array}{ccc} cos\;\beta & 0 & sin\;\beta \\ 0 & 1 & 0\\ -sin\;\beta & 0 & cos\;\beta \end{array}\right]\cdot \left[\begin{array}{ccc} 1 & 0 & 0\\ 0 & cos\;\alpha & -sin\;\alpha \\ 0 & sin\;\alpha & cos\;\alpha \end{array}\right]$$

Among them, $R_{x}(\alpha )$,$R_{y}(\beta )$, and $R_{z}(r)$ are the rotation matrices of the reference coordinate system $Ox_{2}y_{2}z_{2}$ rotating around the $Ox_{2}$ axis, $Oy_{2}^{\prime }$ axis, and Oz axis, respectively.

The three-axis angular velocity output of the gyroscope is:

(4) $$\omega_{1}=R_{1}\cdot \omega +\varepsilon ={\left[\omega_{x_{1}},\omega_{y_{1}},\omega_{z_{1}}\right]}^{T}$$

In Equation (4), ε is the gyro measurement noise, and $R_{1}$ is the rotation matrix from the extinguishing projectile body coordinate system to the gyroscope coordinate system, which is composed of the product of two rotation matrices:

(5) $$R_{1}=R_{y_{1}}(\varphi )\cdot R_{x_{1}}(\theta )\;=\left[\begin{array}{ccc} cos\;\varphi & 0 & sin\;\varphi \\ 0 & 1 & 0\\ -sin\;\varphi & 0 & cos\;\varphi \end{array}\right]\cdot \left[\begin{array}{ccc} 1 & 0 & 0\\ 0 & cos\;\theta & -sin\;\theta \\ 0 & sin\;\theta & cos\;\theta \end{array}\right]$$

Among them, $R_{x_{1}}(\theta )$ and $R_{y_{1}}(\varphi )$ are the rotation matrices of the extinguishing projectile body coordinate system Oxyz rotating around the Ox axis and $Oy_{1}$ axis, respectively. Substituting Equations (1)–(3) and (5) into Equation (4), the angular velocity output expressions of each axis of the gyroscope can be obtained:

(6) $$\begin{array}{c} \omega_{x_{1}}=[cos\;\varphi \cdot cos\;\gamma \cdot cos\;\beta +sin\;\varphi \cdot sin\;\theta \cdot sin\;\gamma \cdot cos\;\beta -sin\;\varphi \cdot cos\;\theta \cdot sin\;\beta ]\omega_{x2}\\ \;+[cos\;\varphi (cos\;\gamma \cdot sin\;\beta \cdot sin\;\alpha -sin\;\gamma \cdot cos\;\alpha )+sin\;\varphi \cdot sin\;\theta (sin\;\gamma \cdot sin\;\beta \cdot sin\;\alpha +cos\;\gamma \cdot cos\;\alpha )\\ \;+sin\;\varphi \cdot cos\;\theta \cdot cos\;\beta \cdot sin\;\alpha ]\omega_{y2}\\ \;+[cos\;\varphi \left(cos\;\gamma \cdot sin\;\beta \cdot cos\;\alpha +sin\;\gamma \cdot sin\;\alpha \right)+sin\;\varphi \cdot sin\;\theta (sin\;\gamma \cdot sin\;\beta \cdot cos\;\alpha -cos\;\gamma \cdot sin\;\alpha )\\ \;+sin\;\varphi \cdot cos\;\theta \cdot cos\;\beta \cdot cos\;\alpha ]\omega_{z2}+\varepsilon \end{array}$$

(7) $$\begin{array}{c} \omega_{y_{1}}=\left[\begin{array}{c} cos\;\theta \cdot sin\;\gamma \cdot cos\;\beta +sin\;\theta \cdot sin\;\beta \end{array}\right]\omega_{x2}\\ \;+\left[cos\;\theta \left(sin\;\gamma \cdot sin\;\beta \cdot sin\;\alpha +cos\;\gamma \cdot cos\;\alpha \right)-sin\;\theta \cdot cos\;\beta \cdot sin\;\alpha \right]\omega_{y2}\\ \;+\left[cos\;\theta \left(sin\;\gamma \cdot sin\;\beta \cdot cos\;\alpha -cos\;\gamma \cdot sin\;\alpha \right)-sin\;\theta \cdot cos\;\beta \cdot cos\;\alpha \right]\omega_{z2}+\varepsilon \end{array}$$

(8) $$\begin{array}{c} \omega_{z_{1}}=\left[-sin\;\varphi \cdot cos\;\gamma \cdot cos\;\beta +cos\;\varphi \cdot sin\;\theta \cdot sin\;\gamma \cdot cos\;\beta -cos\;\varphi \cdot cos\;\theta \cdot sin\;\beta \right]\omega_{x2}\\ \;+[-sin\;\varphi (cos\;\gamma \cdot sin\;\beta \cdot sin\;\alpha -sin\;\gamma \cdot cos\;\alpha )+cos\;\varphi \cdot sin\;\theta (sin\;\gamma \cdot sin\;\beta \cdot sin\;\alpha +cos\;\gamma \cdot cos\;\alpha )\\ \;+cos\;\varphi \cdot cos\;\theta \cdot cos\;\beta \cdot sin\;\alpha ]\omega_{y2}\\ \;+\lfloor -sin\;\varphi \left(cos\;\gamma \cdot sin\;\beta \cdot cos\;\alpha +sin\;\gamma \cdot sin\;\alpha \right)+cos\;\varphi \cdot sin\;\theta \left(sin\;\gamma \cdot sin\;\beta \cdot cos\;\alpha -cos\;\gamma \cdot sin\;\alpha \right)\\ \;+cos\;\varphi \cdot cos\;\theta \cdot cos\;\beta \cdot cos\;\alpha \rfloor \omega_{z2}+\varepsilon \end{array}$$

Extinguishing projectiles are typically fired along the line connecting the gun and the target, with only small lateral deviation; hence, the initial yaw angle and yaw angular rate can be approximated as zero. During the uncontrolled flight phase, a pitch angular velocity inevitably exists due to gravity. However, since this phase is of relatively short duration, the pitch angular velocity can be reasonably approximated as constant, and both pitch and yaw angles are assumed to remain nearly unchanged within this short interval. Under this assumption, the derivation of the roll angle is greatly simplified, and a closed-form solution can be obtained. Moreover, the simplified formulas show that variations in pitch angular velocity have only a negligible effect on the roll angle estimation, confirming that this approximation does not compromise the accuracy or feasibility of the proposed algorithm. Based on the above assumptions, Equations (6)–(8) are simplified to:

(9) $$\begin{array}{c} \omega_{x_{1}}=\left[-cos\;\varphi \cdot sin\;\gamma +\cdot sin\;\varphi \cdot sin\;\theta \cdot cos\;\gamma \right]\omega_{y2}\\ \;+\left[cos\;\varphi \cdot cos\;\gamma \cdot sin\;\beta +sin\;\varphi \cdot sin\;\theta \cdot sin\gamma \cdot sin\;\beta +sin\;\varphi \cdot cos\;\theta \cdot cos\;\beta \right]\omega_{z2}+\varepsilon \end{array}$$

(10) $$\omega_{y_{1}}=\omega_{y2}\cdot cos\;\theta \cdot cos\;\gamma +\omega_{z2}\cdot cos\;\theta \cdot sin\;\beta \cdot sin\;\gamma -\omega_{z2}\cdot sin\;\theta \cdot cos\;\beta +\varepsilon$$

(11) $$\begin{array}{c} \omega_{z_{1}}=\left[\begin{array}{c} sin\;\varphi \cdot sin\;\gamma +cos\;\varphi \cdot sin\;\theta \cdot cos\;\gamma \end{array}\right]\omega_{y2}\\ \;+\left[\begin{array}{c} -sin\;\varphi \cdot cos\;\gamma \cdot sin\;\beta +\cdot cos\;\varphi \cdot sin\;\theta \cdot sin\;\gamma \cdot sin\;\beta +\cdot cos\;\varphi \cdot cos\;\theta \cdot cos\;\beta \end{array}\right]\omega_{z2}+\varepsilon \end{array}$$

During the extinguishing projectile’s flight, the roll angular velocity $\omega_{z2}$ is significantly greater than the pitch angular velocity $\omega_{y2}$. Therefore, when the pitch angle is not zero, $\omega_{z2}\cdot cos\;\theta \cdot sin\;\beta \cdot sin\;\gamma \gg \omega_{y2}\cdot cos\;\theta \cdot cos\;\gamma$, so Equation (10) can be further simplified to:

(12) $$\omega_{y_{1}}=\omega_{z2}\cdot cos\;\theta \cdot sin\;\beta \cdot sin\;\gamma -\omega_{z2}\cdot sin\;\theta \cdot cos\;\beta +\varepsilon$$

In Equation (12), the extinguishing projectile body coordinate system Oxyz and the gyroscope coordinate system $Ox_{1}y_{1}z_{1}$ are fixedly connected, and the θ angle does not change with time. Additionally, the variation in the pitch angle β is extremely small relative to that of the roll angle γ; therefore, $\omega_{z2}\cdot sin\;\theta \cdot cos\;\beta$ only changes in magnitude, not in direction, and this projection appears as a low-frequency DC component. When the extinguishing projectile rolls, the γ angle changes with time, and the change frequency is related to the extinguishing projectile’s roll angular velocity $\omega_{z2}$. So $\omega_{z2}\cdot cos\;\theta \cdot sin\;\beta \cdot sin\;\gamma$ changes periodically with the extinguishing projectile’s roll at an angular frequency, appearing as a high-frequency AC component.

## 3. Data Filtering and Roll Attitude Calculation

### 3.1. Data Filtering

Since the γ angle changes with the rotation of the extinguishing projectile, during filtering, the component matching the frequency corresponding to the current roll angular velocity (i.e., the component related to the roll angle γ) is retained, and other frequency components are filtered out.

In real-time signal processing systems, band-pass filters are often used to extract signals in specific frequency bands. Traditional fixed-coefficient filters are difficult to cope with scenarios where the center frequency changes dynamically. To achieve effective separation of high-frequency pitch components, this paper adopts a Dynamic Coefficient Biquad Band-pass Filter. By updating the filter parameters in real-time, it realizes dynamic tracking of the center frequency, which is particularly suitable for scenarios requiring rapid response to frequency changes. Its transfer function is expressed as a time-varying system:

(13) $$H(z,n)=\frac{b_{0}(n)+b_{2}(n)z^{-2}}{1+a_{1}(n)z^{-1}+a_{2}(n)z^{-2}}$$

The coefficients in Equation (13) are dynamically updated according to the instantaneous frequency $f_{c}(t)$:

(14) $$\left\{\begin{array}{c} \omega_{0}(n)=2\pi f_{c}(n)/f_{s}\\ \alpha (n)=sin\;\omega_{0}(n)/(2Q)\\ b_{0}(n)=\frac{sin\;\omega_{0}(n)}{2(1+\alpha (n))}\\ b_{2}(n)=-b_{0}(n)\\ a_{1}(n)=-2cos\;\omega_{0}(n)/(1+\alpha (n))\\ a_{2}(n)=(1-\alpha (n))/(1+\alpha (n)) \end{array}\right.$$

Among them, $\omega_{0}(n)$, is the discrete-domain center angular frequency, $f_{s}$ is the sampling frequency, and Q is the quality factor. The filter is implemented using a direct II-type structure, and the calculation is completed with minimal memory through a difference equation. The relationship between its state update and output is as follows:

(15) $$\left\{\begin{array}{c} \omega_{0}[n]=x[n]-a_{1}(n)\omega_{1}[n-1]\\ -a_{2}(n)\omega_{2}[n-1]\\ y[n]=b_{0}(n)\omega_{0}[n]+b_{2}(n)\omega_{2}[n-1]\\ \omega_{1}[n]=\omega_{0}[n]\\ \omega_{2}[n]=\omega_{1}[n-1] \end{array}\right.$$

In Equation (15), x[n] is the input signal, y[n] is the output signal, and $\omega_{1}[n]$ and $\omega_{2}[n]$ are the state variables of the filter, used to store the intermediate values of the delay unit.

In practical applications, the discrete-domain center angular frequency satisfies:

(16) $$\omega_{0}\left(n\right)={|\omega }_{z}\left(n\right)|/360$$

During the extinguishing projectile’s flight, the roll angular velocity $\omega_{z}$ is significantly greater than the pitch and yaw angular velocities, i.e., $\omega_{z}\gg \omega_{x},\;\omega_{y}$. Thus, the coupling effect of lateral angular velocities on the roll component can be ignored. The magnitude of the extinguishing projectile’s roll angular velocity is approximately estimated by calculating the modulus of the three-axis output data of the gyroscope:

(17) $$|\omega_{z}|\approx \sqrt{\omega_{x_{1}}^{2}+\omega_{y_{1}}^{2}+\omega_{z_{1}}^{2}}$$

To suppress the gyro random walk and bias instability noise, a moving average process is performed on the estimated actual roll angular velocity of the extinguishing projectile:

(18) $${\bar{\omega }}_{z}(k)=\frac{1}{N(k)}\sum_{i=0}^{N-1}\;\omega_{z}(k-i)$$

The window length  N  is dynamically adjusted according to the roll angular velocity:

(19) $$N(k)=round(\frac{\alpha \cdot f_{s}}{\left|\frac{\mathrm{d}\omega_{z}}{dt}\right|+\varepsilon })$$

The parameter settings are as follows:

α: Sensitivity coefficient (typical value: 0.1~0.3);
fs: Sampling frequency (100–1000 Hz);
ε: Minimum value (to avoid division by zero, 1 × 10^−6^).

The filter design utilizes instantaneous frequency information, and realizes accurate tracking of the center frequency through the calculated system coefficients. During the air alignment process of the firefighting extinguishing projectile, due to the change in the flight roll angular velocity, the high-frequency component will have a frequency offset. The use of the dynamic coefficient second-order IIR band-pass filter can effectively cope with this frequency change, ensuring accurate extraction and processing of the signal.

Additionally, this filter exhibits excellent stability and robustness. In practical applications, the performance of the band-pass filter may be affected by the external environmental noise and interference. However, by dynamically updating the filter parameters, the filter can adapt to different working environments, thereby ensuring the stability and reliability of the filtering performance.

After filtering, the high-frequency components can be effectively extracted, significantly improving the signal-to-noise ratio (SNR). This not only contributes to improve the attitude calculation accuracy of the firefighting extinguishing projectile, but also enhances the anti-interference ability of the entire guidance system. This dynamic-coefficient filter structure generalizes to other adaptive filtering problems, providing a theoretical contribution to time-varying frequency estimation.

### 3.2. Roll Attitude Calculation

When periodic peaks appear in the filtered signal, it indicates that the direction of the $Oy_{1}$ axis of the gyroscope is orthogonal to the extinguishing projectile’s pitch plane at that moment (i.e., instantaneously aligned with the pitch direction). The real roll attitude angle can be calculated by combining time information.

Envelope detection is performed on the high-pass filtered signal $\omega_{\alpha }^{\prime \prime }$ to extract the periodic peak moments $t_{k}$. The roll attitude angle is corrected by integrating ${\bar{\omega }}_{z}$ over time and combining the peak phase:

(20) $$\phi (t)=\int_{0}^{t}\;{\bar{\omega }}_{z}dt+\sum_{k}\;\Delta \phi_{k}\cdot \delta (t-t_{k})$$

Among them, $\Delta \phi_{k}$ is the phase correction amount corresponding to the k -th peak.

## 4. Experimental Results and Analysis

In this section, simulations and experiments are conducted to verify the feasibility and effectiveness of the method proposed in this paper, and the relevant verification process is carried out in accordance with the method shown in Figure 3.

### 4.1. Modeling and Verification of Projectile External Ballistics Simulation Based on MATLAB

To meet the requirements of external ballistic characteristic analysis and attitude calculation of high-speed rotating extinguishing projectiles, this paper designs a numerical simulation framework based on Newton–Euler equations, which realizes the prediction of 3D trajectory and the simulation of roll attitude evolution, and generates gyroscope virtual measurement data containing noise, providing a high-confidence simulation environment for the verification of on-board attitude estimation algorithms.

To describe the rotational motion of the projectile, the angular rate vector is expressed in the body coordinate system, with $\omega_{B}=[p\;q\;r{]}^{⊤}$ denoting the angular rate vector about the body axes (x,y,z) and $I_{B}=diag(I_{x},I_{y},I_{z})$ being the inertia tensor. The rotational motion is governed by

(21) $$I_{B}{\dot{\omega }}_{B}+\omega_{B}\times (I_{B}\omega_{B})=\tau_{B}$$

which can be expanded as

(22) $$\left\{\begin{array}{c} \dot{p}=\frac{I_{y}-I_{z}}{I_{x}}qr+\frac{\tau_{x}}{I_{x}},\\ \dot{q}=\frac{I_{z}-I_{x}}{I_{y}}rp+\frac{\tau_{y}}{I_{y}},\\ \dot{r}=\frac{I_{x}-I_{y}}{I_{z}}pq+\frac{\tau_{z}}{I_{z}}. \end{array}\right.$$

The body torques consist of aerodynamic damping and launch-induced disturbances:

(23) $$\tau_{B}=[-c_{r}p,-c_{p}q,-c_{y}r{]}^{⊤}+\delta \tau (t)$$

enabling realistic simulation of roll-dominant motion.

The simulation initial conditions are set as shown in Table 1.

**Figure 4:**
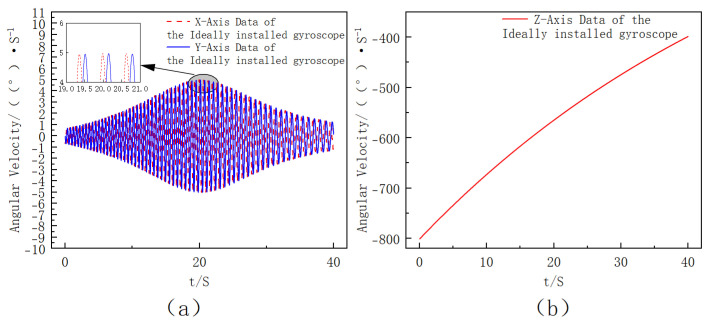
Ideal three-axis angular rate outputs under perfect alignment between the gyroscope and projectile body axes ((**a**) *X*- and *Y*-axis angular velocity data of the ideally installed gyroscope, where the inset shows a partial enlargement. (**b**) Z-axis angular velocity data of the ideally installed gyroscope.).

Under the ideal condition where the gyroscope axes are perfectly aligned with the projectile body axes, the gyroscope outputs can be directly used for attitude computation. The ideal angular rate data under this perfect alignment assumption are shown in Figure 4. The roll-rate range of approximately 1–2 rot/s is selected according to the typical uncontrolled flight characteristics of mortar-type extinguishing projectiles, where the roll motion is mainly induced by initial disturbances rather than aerodynamic spin stabilization.

**Figure 5:**
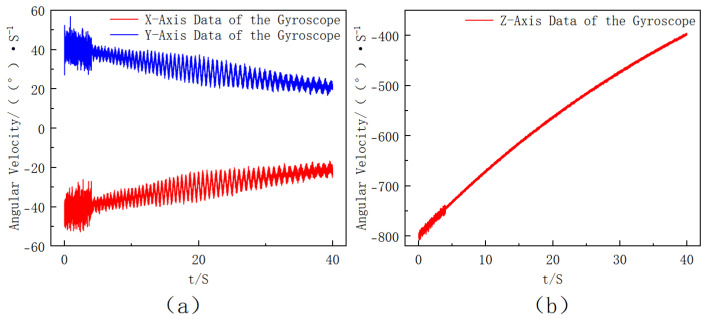
Curves of Three-Axis Angular Velocity of the Gyroscope with Time (this figure presents the simulated output of the MEMS gyroscope including sensor noise and bias drift. (**a**) *X*- and *Y*-axis angular velocity data of the gyroscope. (**b**) Z-axis angular velocity data of the gyroscope.).

The simulation results indicate that the roll angular rate exhibits an exponential decay over time due to aerodynamic damping, representing the typical roll-dominant rotational behavior of the extinguishing projectile. Considering the existence of installation error angles, it is necessary to perform axis rotation correction on the three-axis data. To ensure consistency with the experiments, the noise model was constructed according to the specifications of the ICM-20649 gyroscope. According to the datasheet, the gyro noise density is about 0.0017°/s/√Hz, which corresponds to an angle random walk of ~0.2°/√s at the simulation sampling rate; the bias stability is approximately ±3°/s; and the scale factor error is set to 1%. In the MATLAB R2021a simulation, these error sources together with a 3° installation misalignment were incorporated into the synthetic measurements. Furthermore, a larger bias disturbance was applied during the first 2 s to emulate the unstable state of the sensor immediately after power-up as well as launch-induced jitter. This model provides a realistic representation of the ICM-20649 measurement characteristics, the obtained three-axis angular velocity data of the gyroscope are shown in Figure 5.

**Figure 6:**
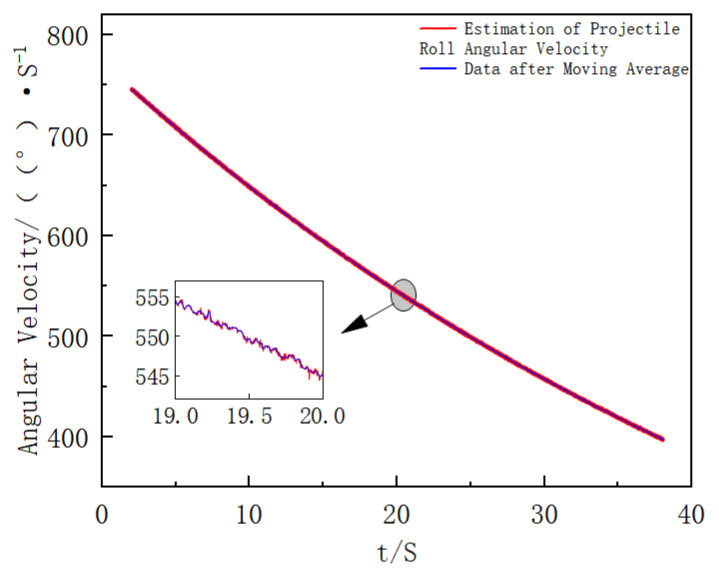
Estimated Actual Roll Angular Velocity of the Projectile and Moving Average Result (the figure shows the estimated roll angular velocity and its moving-average result used to derive the adaptive filter frequency).

**Figure 7:**
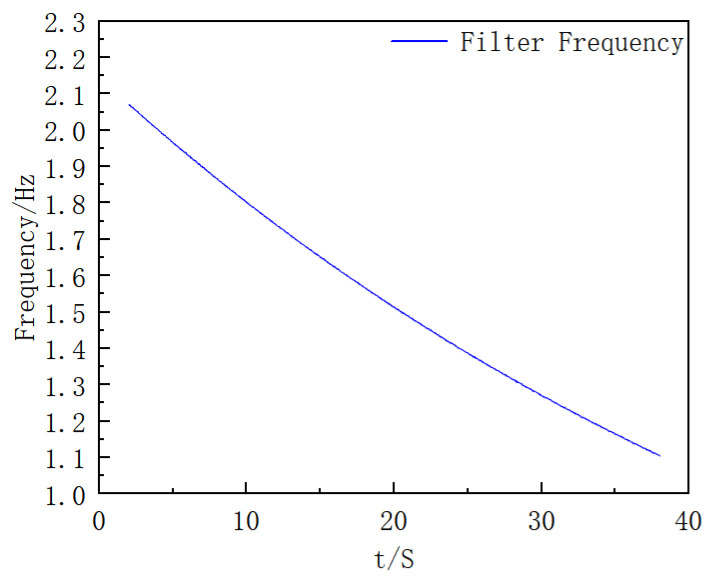
Filter Frequency Curve with Time (this figure illustrates the time-varying center frequency of the adaptive IIR filter during operation).

In the simulation results, the data fluctuates significantly and has poor stability at the initial stage of extinguishing projectile launch due to the influence of extinguishing projectile fluctuation. The processed data is closer to the actual extinguishing projectile attitude information during flight. The data after 2 s is selected for analysis, and the modulus of the three-axis data of the gyroscope is calculated to estimate the actual roll angular velocity of the extinguishing projectile. The results are shown in Figure 6. By combining the data after moving average processing in Figure 6, the angular velocity is divided by 360 to further calculate the filtered frequency, and the result is shown in Figure 7.

**Figure 8:**
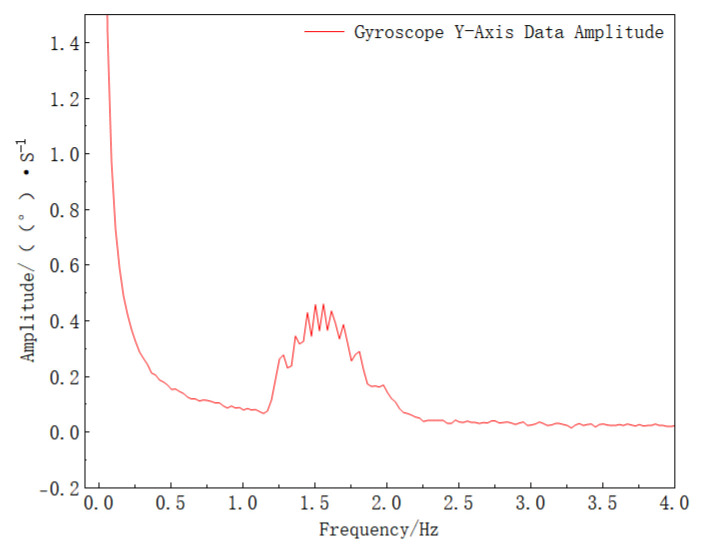
Spectrum of the gyroscope *Y*-axis signal (this spectrum reveals the dominant frequency component in the *Y*-axis gyroscope signal used for filter tuning).

To separate different frequency components in the signal, Fourier transform is performed on the gyro *Y*-axis data, and the results are shown in Figure 8.

**Figure 9:**
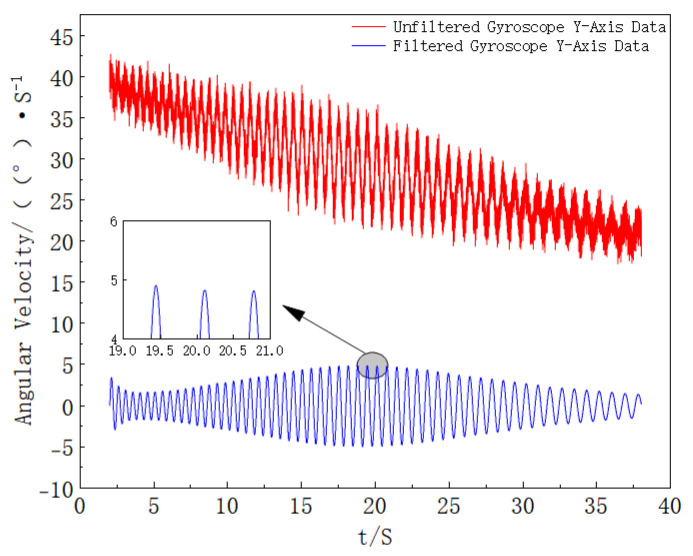
Comparison of Gyro *Y*-axis Data Before and After Filtering (The comparison verifies that the dynamic IIR filter effectively suppresses noise and preserves periodic characteristics).

As clearly observed in Figure 8, a fixed low-frequency signal and a varying high-frequency signal are present, which is consistent with the theoretical analysis, verifying the feasibility of the proposed method. To extract the target frequency signal, the signal is processed using a dynamic coefficient second-order IIR band-pass filter, and the filtering results are shown in Figure 9.

**Figure 10:**
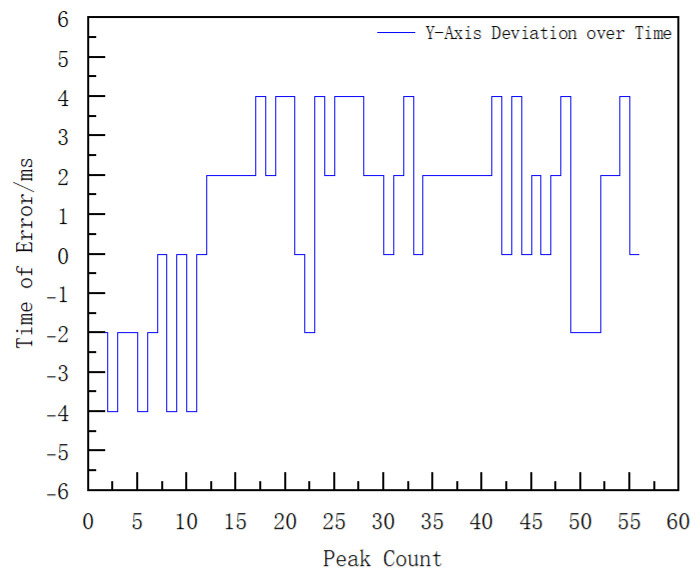
Error of Peak Moments (This figure shows the difference between the peak moments of the projectile and gyroscope signals after filtering).

It can be seen from Figure 9 that the filtered data shows smooth periodic changes. To further analyze the signal characteristics, envelope detection is performed on the data in Figure 9 to calculate the peak moments. At the same time, envelope detection is also performed on the *Y*-axis of the original data to calculate the peak moments. The error of the peak moments is obtained through the difference between the two sets of data, and the results are shown in Figure 10.

**Figure 11:**
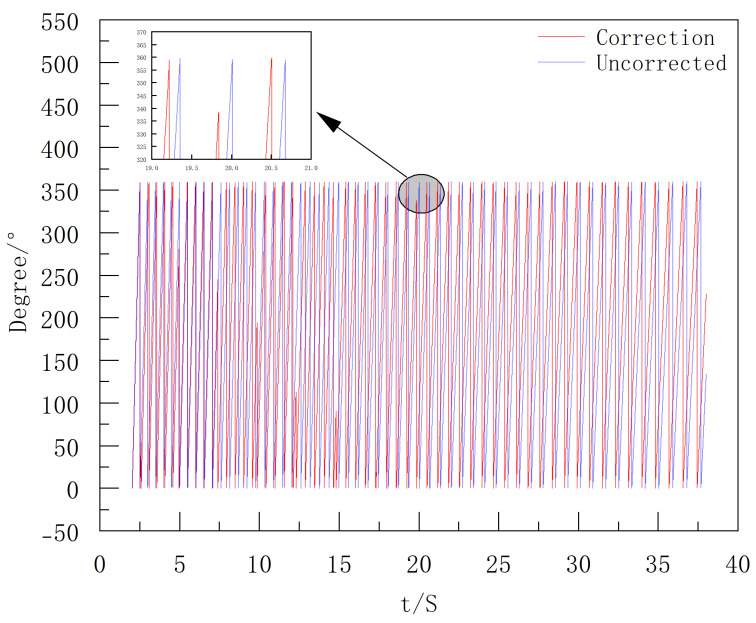
Comparison of roll attitude angles before and after simulation data correction.

Figure 10 shows that the time difference between peak positions is within 4 ms, and the angle calculation error remains below 2.68°. Based on the above analysis, the roll attitude angle is corrected by integrating $w_{z}$ over time and combining the peak-time adjustment. The comparison of the roll attitude angles before and after correction is presented in Figure 11.

### 4.2. Turntable Experiment

**Figure 12:**
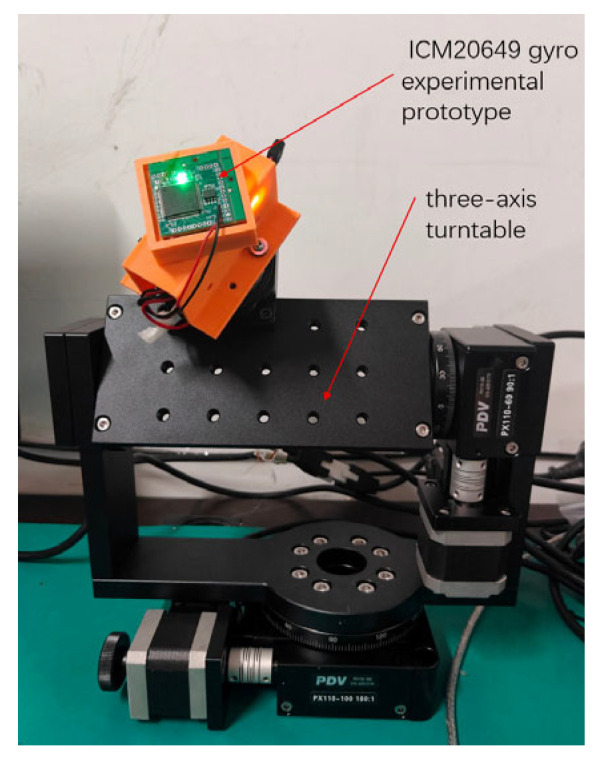
Experimental Platform (the photo displays the experimental turntable setup used to validate the proposed algorithm).

The experimental setup consists of an ICM20649 gyro experimental prototype, three-axis turntable, turntable control system, and power supply. The ICM20649 gyro experimental prototype is installed on the three-axis turntable with intentionally introduced misalignment angles between the gyroscope’s axes and the turntable’s axes to better simulate real-world application scenarios. The experimental data is stored in the Flash of the main control chip of the experimental prototype, and the experimental platform is shown in Figure 12.

**Figure 13:**
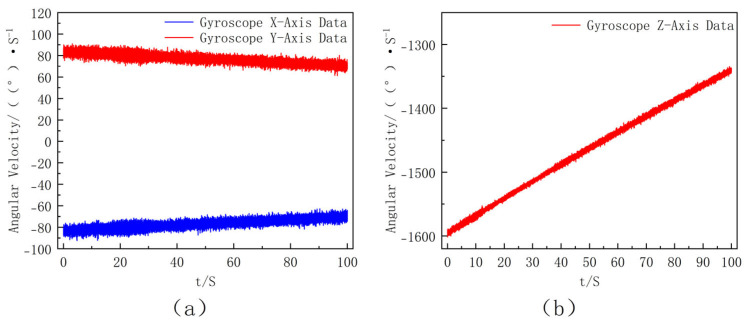
Three-Axis Output of the Gyroscope (this figure presents the three-axis gyroscope outputs acquired during the turntable experiment, which serve as the measured inputs for subsequent frequency estimation and filtering. (**a**) *X*- and *Y*-axis angular velocity data of the gyroscope. (**b**) Z-axis angular velocity data of the gyroscope.).

The dynamic experimental process is consistent with the aforementioned simulation. The initial roll angular velocity of the turntable is set to 1600 dps, the data acquisition frequency is 200 Hz, the gyro range is selected as ±2000 dps, and a total of 20,000 sets of data samples are collected; the pitch angular velocity oscillates sinusoidally with its amplitude linearly increasing from 0.5°/s to 1°/s over time, while the yaw angular velocity oscillates sinusoidally around 0°/s with a fixed amplitude of approximately ±1°/s. This experimental setup introduced dynamic variations in both pitch and yaw while maintaining controllability, making the conditions closer to real flight scenarios and allowing for a more comprehensive evaluation of the robustness of the proposed algorithm. The output data of the gyroscope are shown in Figure 13.

**Figure 14:**
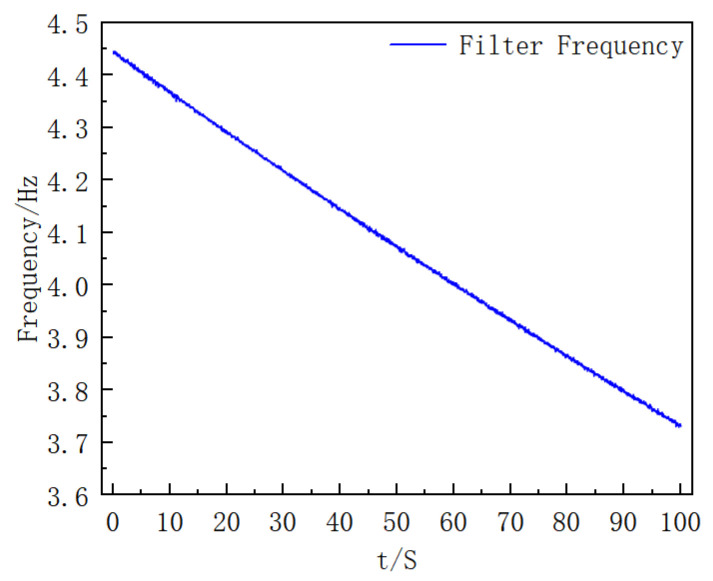
Filter Frequency Curve with Time (This figure shows the time evolution of the adaptive band-pass filter’s center frequency computed from the smoothed roll-rate estimate, consistent with the method description).

Consistent with the data processing idea in the simulation process, the modulus of the three-axis angular velocity of the gyroscope is first calculated to obtain the estimated roll angular velocity of the extinguishing projectile; then, moving average processing is performed on it, and finally, the filter frequency is solved. The results are shown in Figure 14.

**Figure 15:**
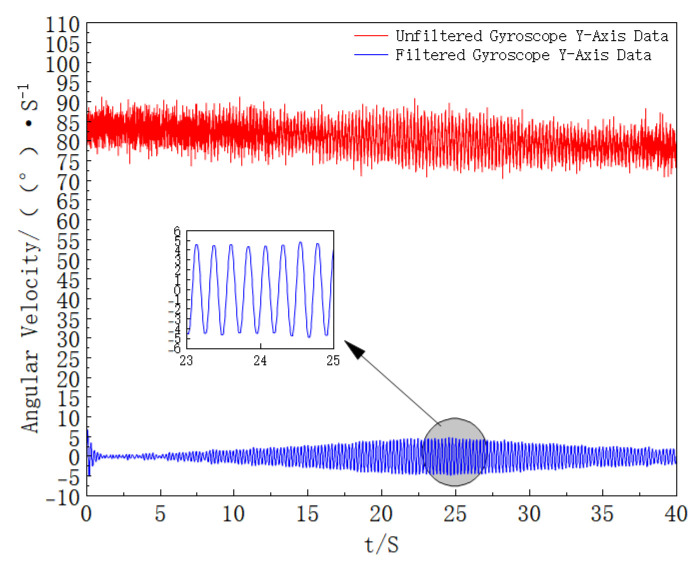
Comparison of *Y*-axis Data Before and After Processing (the comparison demonstrates that the dynamic IIR filtering suppresses high-frequency noise in the *Y*-axis signal while preserving the roll-related component).

The *Y*-axis data of the gyroscope is filtered, and the stable data in the middle period is selected for analysis. The comparison results of the *Y*-axis data before and after processing are shown in Figure 15.

**Figure 16:**
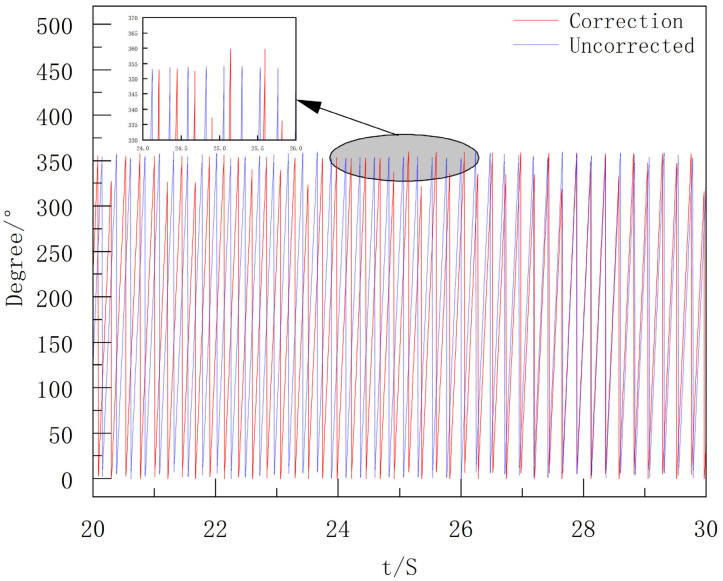
Comparison of Roll Attitude Angles Before and After Correction.

After obtaining the filtered *Y*-axis data, envelope detection is performed to extract its peak moments, which are used as the basis to correct the integral process of the extinguishing projectile’s roll angular velocity. The final roll angle results are shown in Figure 16.

### 4.3. Result Analysis

The experimental results demonstrate that the time difference between the signal peak moments of the extinguishing projectile *Y* axis and the gyroscope *Y*-axis is ≤4 ms, and the maximum roll angle error is ≤2.68°. These results are within the engineering requirement of ≤5 ms synchronization error under high-dynamic conditions. This indicates that the proposed method achieves the required dynamic synchronization performance.

More importantly, the error-compensated roll attitude provides a reliable reference for real-time projectile attitude estimation. Compared with conventional multi-sensor fusion schemes, the single-gyro architecture demonstrates several advantages. First, it significantly reduces hardware complexity and cost while ensuring comparable accuracy. Second, it simplifies the controller’s computational load, making the method more suitable for real-time embedded applications. These results confirm both the engineering feasibility and the practical economic value of the proposed method.

Nevertheless, certain limitations should be acknowledged. For example, the evaluation was conducted under controlled conditions, and further verification is required in environments with extreme disturbances, such as high overload, thermal stress, or electromagnetic interference. Additionally, while the method effectively suppresses noise and frequency drift, its long-term stability in complex battlefield conditions remains to be fully demonstrated. These observations provide directions for future refinement and validation.

## 5. Conclusions

This paper proposed a novel roll angle estimation method based on a single gyroscope, tailored for precision firefighting projectiles operating in high-speed roll environments. By introducing the framework of “modulus estimation–phase decoupling–dynamic filtering”, the study effectively addressed the limitations of traditional multi-sensor fusion schemes, including structural complexity and high cost. In particular, the application of a dynamic-coefficient second-order IIR band-pass filter enabled accurate tracking of time-varying roll frequencies, substantially improving pitch component separation accuracy.

The dual validation through ballistic simulations and turntable experiments confirmed the effectiveness and real-time feasibility of the method, with a maximum roll angle error below 3%, phase detection error within 4 ms, and execution time of 0.5 ms on an MCU platform. These results demonstrate that the proposed method meets the stringent real-time requirements of onboard systems while reducing system complexity and cost.

The main contribution of this research lies not only in providing a cost-effective engineering solution but also in advancing the methodological foundation of single-sensor navigation under high-dynamic conditions. Theoretically, the proposed approach extends the scope of attitude estimation methods by demonstrating that accurate roll angle extraction can be achieved with minimal sensor architecture. Practically, it offers a scalable and economical pathway for large-scale deployment. Conventional multi-sensor fusion modules typically require a gyroscope, a accelerometer, a magnetometer, and an auxiliary GNSS receiver, with a total hardware cost of around 90–100 USD. In contrast, the proposed single-gyro system relies only on one MEMS gyroscope (ICM-20649) and a simplified processing circuit, with an approximate cost of 35 USD. This corresponds to a cost reduction of nearly 60%, while maintaining comparable estimation accuracy and robustness.

Future research will address key challenges such as bias compensation under extreme overloads, multi-physics coupling error modeling in harsh operational environments, and real-time trajectory identification tightly integrated with attitude estimation. By tackling these issues, this study lays both a theoretical and practical foundation for the development of lightweight, autonomous navigation technologies for precision firefighting projectiles and similar high-dynamic platforms, ultimately contributing to enhanced system reliability, reduced costs, and improved firefighting effectiveness.

## Figures and Tables

**Table 1:** Initial simulation and filtering parameter settings.

Parameter	Value/Setting
Initial velocity	600 m/s
Initial pitch angle	45°
Initial roll/yaw angle	0°/0°
Initial roll angular rate	800°/s
Damping coefficient	1°/s
Simulation duration	40 s
Time step	0.01 s
Q (quality factor)	4
α (Sensitivity coefficient)	0.2

## Data Availability

Data available upon request.
